# Supplementary material for: Antibiotic potentiation and inhibition of cross-resistance in pathogens associated with cystic fibrosis
Source: bioRxiv. 2025 Jun 2:2023.08.02.551661. Originally published 2023 Aug 2. Preprint. [Version 2] doi: 10.1101/2023.08.02.551661 (PMC10418187; doi:10.1101/2023.08.02.551661)
Supplement: Supplement 1 [file NIHPP2023.08.02.551661v2-supplement-1.pdf]

bioRxiv preprint doi: https://doi.org/10.1101/2023.08.02.551661; this version posted June 2, 2025. The copyright holder for this preprint (which was not certified by peer review) is the author/funder, who has granted bioRxiv a license to display the preprint in perpetuity. It is made available under aCC-BY 4.0 International license.

# SUPPLEMENTARY INFORMATION FOR

## Antibiotic potentiation and inhibition of cross-resistance in pathogens associated with cystic fibrosis

Nikol Kadeřábková[1,3,†], R. Christopher D. Furniss[3,†], Evgenia Maslova[4], Kathryn E. Potter[1], Lara Eisaiankhongi[4], Patricia Bernal[5], Alain Filloux[3,6,7,8], Cristina Landeta[9], Diego Gonzalez[10], Ronan R. McCarthy[4], Despoina A.I. Mavridou[1,2*]

[1]Department of Molecular Biosciences, The University of Texas at Austin, Austin, 78712, Texas, USA
[2]John Ring LaMontagne Center for Infectious Diseases, The University of Texas at Austin, Austin, 78712, Texas, USA
[3]Centre for Bacterial Resistance Biology, Department of Life Sciences, Imperial College London, London, SW7 2AZ, UK
[4]Division of Biosciences, Department of Life Sciences, College of Health and Life Sciences, Brunel University London, Uxbridge, UB8 3PH, UK
[5]Departamento de Microbiología, Facultad de Biología, Universidad de Sevilla, Seville, 41012, Spain
[6]Singapore Centre for Environmental Life Sciences Engineering, Nanyang Technological University, 637551, Singapore
[7]School of Biological Sciences, Nanyang Technological University, 639798, Singapore
[8]Lee Kon Chian School of Medicine, Nanyang Technological University, 636921, Singapore
[9]Department of Biology, Indiana University, Bloomington, Indiana, 47405, USA.
[10]Laboratoire de Microbiologie, Institut de Biologie, Université de Neuchâtel, Neuchâtel, 2000, Switzerland

[*]Correspondence: despoina.mavridou@austin.utexas.edu
[†]These authors have contributed equally to this work

**This PDF file includes:**

Figures S1 to S5
Tables S1 to S5
Legends for Files S1 to S5
Supplementary references

bioRxiv preprint doi: https://doi.org/10.1101/2023.08.02.551661; this version posted June 2, 2025. The copyright holder for this preprint (which was not certified by peer review) is the author/funder, who has granted bioRxiv a license to display the preprint in perpetuity. It is made available under aCC-BY 4.0 International license.

## SUPPLEMENTARY FIGURES

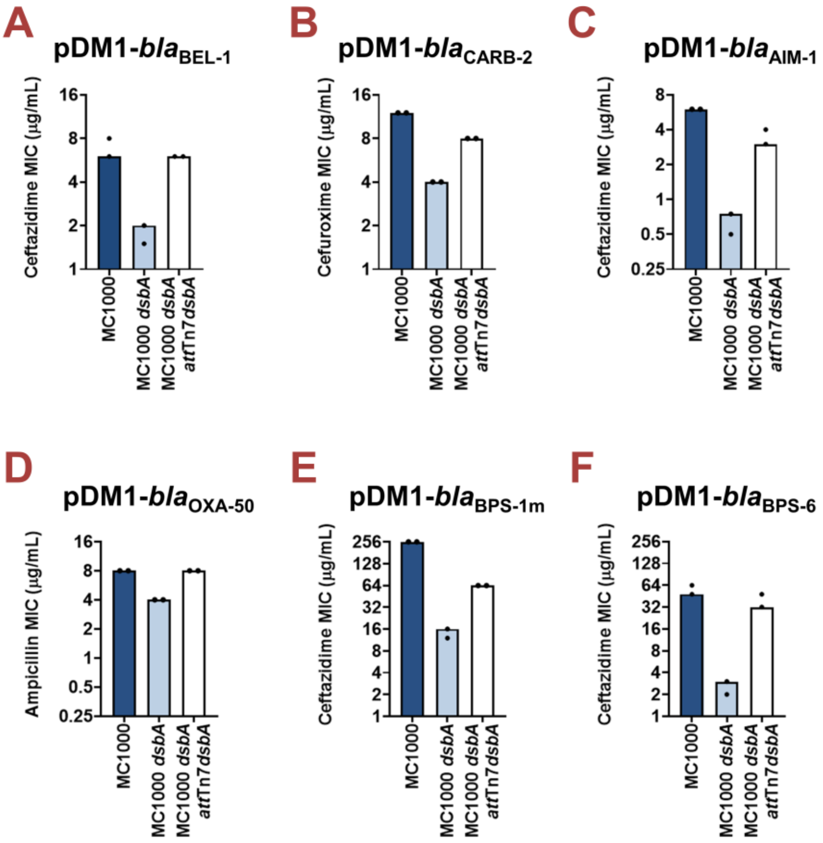


**Figure S1. Complementation of *dsbA* restores the β-lactam MIC values for *E. coli* MC1000 *dsbA* expressing β-lactamase enzymes.** Re-insertion of *dsbA* at the *att*Tn*7* site of the chromosome restores representative β-lactam MIC values for *E. coli* MC1000 *dsbA* harboring **(A)** pDM1-*bla*$_{\text{BEL-1}}$ (ceftazidime MIC), **(B)** pDM1-*bla*$_{\text{CARB-2}}$ (cefuroxime MIC), **(C)** pDM1-*bla*$_{\text{AIM-1}}$ (ceftazidime MIC), **(D)** pDM1-*bla*$_{\text{OXA-50}}$ (ampicillin MIC), **(E)** pDM1-*bla*$_{\text{BPS-1m}}$ (ceftazidime MIC), and **(F)** pDM1-*bla*$_{\text{BPS-6}}$ (ceftazidime MIC). Graphs show MIC values (μg/mL) and are representative of two biological experiments, each conducted as a single technical repeat.

bioRxiv preprint doi: https://doi.org/10.1101/2023.08.02.551661; this version posted June 2, 2025. The copyright holder for this preprint (which was not certified by peer review) is the author/funder, who has granted bioRxiv a license to display the preprint in perpetuity. It is made available under aCC-BY 4.0 International license.

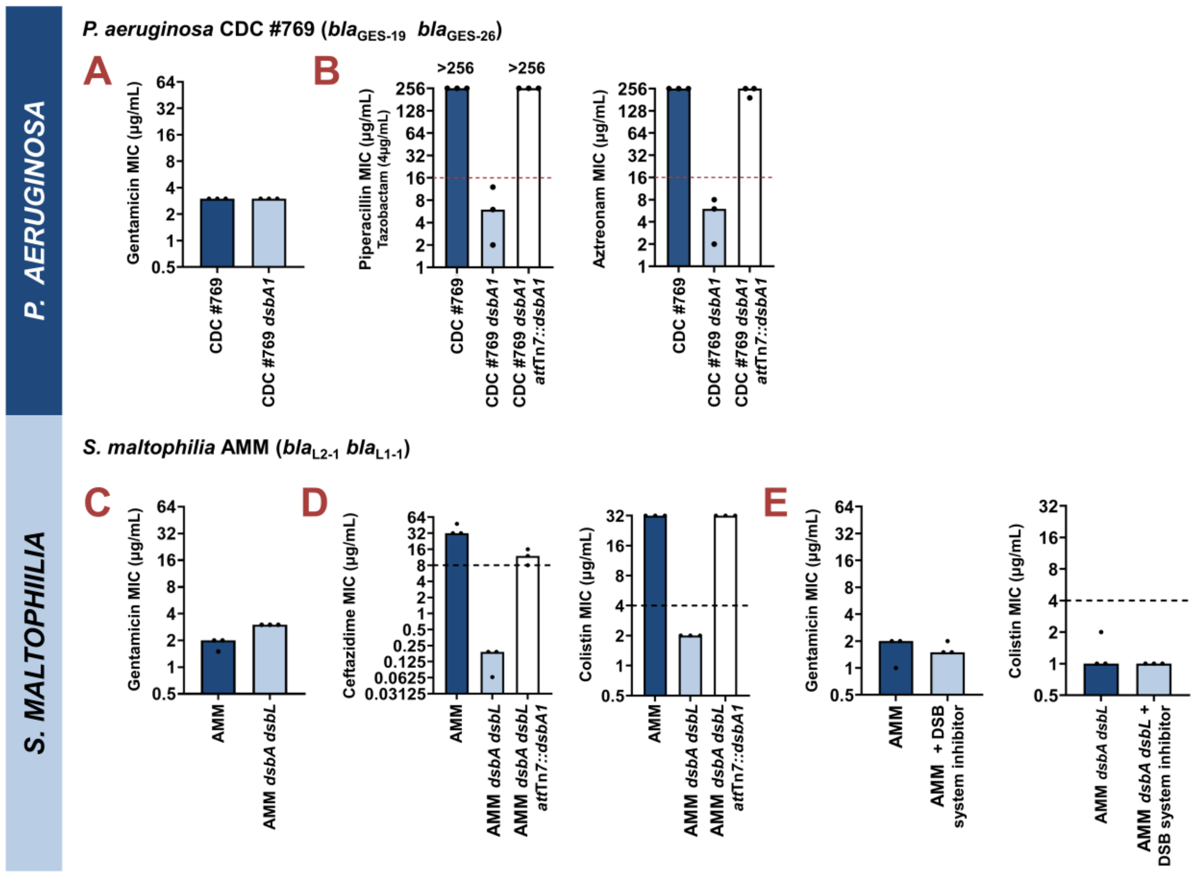


**Figure S2. Assessment of off-target effects for clinical strains of *P. aeruginosa* and *S. maltophilia* that are deficient in oxidative protein folding. (A)** *P. aeruginosa* CDC #769 and its mutant lacking *dsbA1* have identical gentamicin MIC values, confirming that absence of DsbA does not compromise the general ability of the strain to resist antibiotic stress. **(B)** Re-insertion of the *dsbA1* gene from *P. aeruginosa* PAO1 at the *att*Tn*7* site of the chromosome restores representative antibiotic MIC values for *P. aeruginosa* CDC #769 *dsbA1* (left, piperacillin/tazobactam MIC; right, aztreonam MIC). **(C)** *S. maltophilia* AMM and its mutant lacking *dsbA* and *dsbL* have near-identical gentamicin MIC values, confirming that absence of DsbA and DsbL does not compromise the general ability of the strain to resist antibiotic stress. **(D)** Re-insertion of the *dsbA1* gene from *P. aeruginosa* PAO1 at the *att*Tn*7* site of the chromosome restores representative antibiotic MIC values for *S. maltophilia* AMM *dsbA dsbL* (left, ceftazidime MIC; right, colistin MIC). **(E)** Changes in MIC values observed using the DSB system inhibitor (compound 36) are due solely to inhibition of the DSB system. The gentamicin MIC value of *S. maltophilia* AMM remains unchanged upon addition of the inhibitor (left), and the same is observed for the colistin MIC value of *S. maltophilia* AMM *dsbA dsbL* in the presence of the compound (right). This indicates that the chemical compound used in this study only affects the function of the DSB system proteins. For all panels, graphs show MIC values (µg/mL) and are representative of three biological experiments. β-Lactam MICs were conducted as a single technical repeat and colistin MICs were conducted in technical triplicate; red dotted lines indicate the EUCAST clinical breakpoint for each antibiotic, where applicable. In the absence of EUCAST clinical breakpoints for *S. maltophilia*, the black dotted lines indicate the EUCAST clinical breakpoint for each antibiotic for the related pathogen *P. aeruginosa*, where applicable.

bioRxiv preprint doi: https://doi.org/10.1101/2023.08.02.551661; this version posted June 2, 2025. The copyright holder for this preprint (which was not certified by peer review) is the author/funder, who has granted bioRxiv a license to display the preprint in perpetuity. It is made available under aCC-BY 4.0 International license.

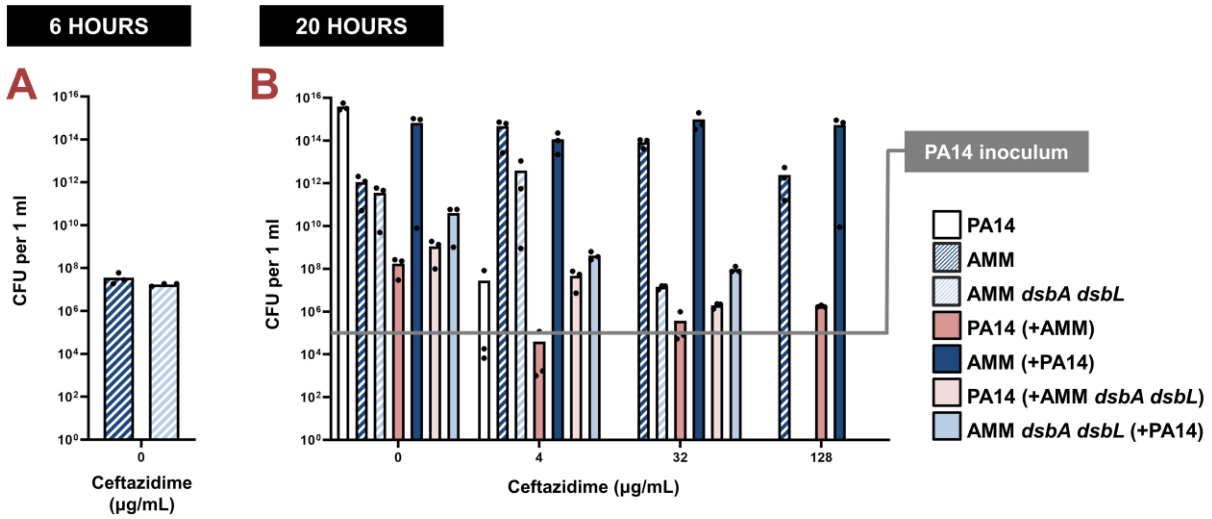


**Figure S3. Protection of *P. aeruginosa* by *S. maltophilia* clinical strains is dependent on oxidative protein folding. (A)** Comparison of the colony forming units (CFUs) of *S. maltophilia* AMM with the CFUs of *S. maltophilia* AMM *dsbA dsbL* after six hours of growth, prior to *P. aeruginosa* PA14 addition. The two *S. maltophilia* strains display equivalent growth. **(B)** Complementary analysis to Fig. 4E; here the CFUs of all *P. aeruginosa* and *S. maltophilia* strains were enumerated in isolation and in mixed culture conditions for a more limited set of antibiotic concentrations. Equivalent trends to Fig. 4E are observed. The susceptible *P. aeruginosa* strain PA14 can survive exposure to ceftazidime up to a maximum concentration of 4 µg/mL when cultured in isolation (white bars). By contrast, if co-cultured in the presence of *S. maltophilia* AMM (dark blue bars), which can hydrolyze ceftazidime through the action of its L1-1 β-lactamase enzyme, *P. aeruginosa* PA14 (dark pink bars) can survive and actively grow in higher concentrations of ceftazidime (see 128 µg/mL of ceftazidime). This protection is abolished if *P. aeruginosa* PA14 (light pink bars) is co-cultured with *S. maltophilia* AMM *dsbA dsbL* (light blue bars). In this case, L1-1 is inactive (as shown in Fig. 4AB and [1]), resulting in killing of *S. maltophilia* AMM and, in turn, eradication of *P. aeruginosa* PA14 (see 128 µg/mL of ceftazidime, absence of light pink bars). Three biological replicates were conducted in technical triplicate and mean CFU values are shown. The grey line indicates the *P. aeruginosa* PA14 inoculum. The mean CFU values used to generate this figure are presented in File S2C.

bioRxiv preprint doi: https://doi.org/10.1101/2023.08.02.551661; this version posted June 2, 2025. The copyright holder for this preprint (which was not certified by peer review) is the author/funder, who has granted bioRxiv a license to display the preprint in perpetuity. It is made available under aCC-BY 4.0 International license.

A

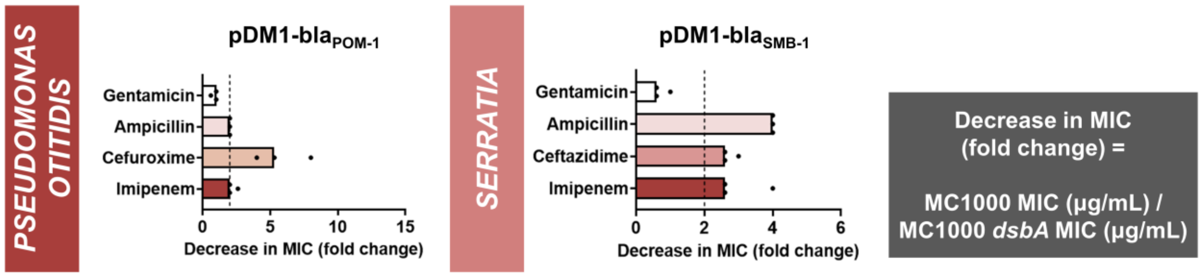


B

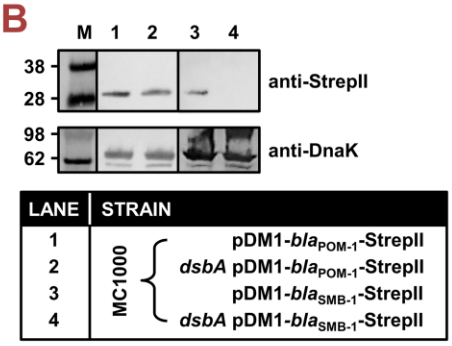


C

| Strain (MC1000) | Enzyme stability | Nitrocefin hydrolysis† |
|---|---|---|
| pDM1 | vector control | 1.82 ± 3.20 |
| *dsbA* pDM1 | vector control | 0.96 ± 2.06$^{ns}$ |
| pDM1-*bla*$_{L2-1}$ | disulfide independent | 82.66 ± 1.26 |
| *dsbA* pDM1-*bla*$_{L2-1}$ | disulfide independent | 82.99 ± 0.77$^{ns}$ |
| pDM1-*bla*$_{POM-1}$ | not degrading | 27.87 ± 1.70 |
| *dsbA* pDM1-*bla*$_{POM-1}$ | not degrading | 22.23 ± 1.67* |
| pDM1-*bla*$_{SMB-1}$ | degrading | 144.96 ± 14.69 |
| *dsbA* pDM1-*bla*$_{SMB-1}$ | degrading | 12.11 ± 4.33* |

†nM.mg$^{-1}$ pellet.15 min$^{-1}$

**Figure S4. The activity of additional species-specific β-lactamases depends on disulfide bond formation. (A)** β-Lactam MIC values for *E. coli* MC1000 expressing disulfide-bond-containing β-lactamases from *P. otitidis* (left, POM-1; Table S1) and *Serratia spp*. (right, SMB-1; Table S1) are reduced in the absence of DsbA (MIC fold changes: >2; fold change of 2 is indicated by the black dotted lines). No changes in MIC values are observed for the aminoglycoside antibiotic gentamicin (white bars) confirming that absence of DsbA does not compromise the general ability of this strain to resist antibiotic stress. Graphs show MIC fold changes for β-lactamase-expressing *E. coli* MC1000 and its *dsbA* mutant. MIC assays were performed in three biological experiments each conducted as a single technical repeat; the MIC values used to generate this figure are presented in File S2A (rows 22-25). **(B)** Protein levels of disulfide-bond-containing β-lactamases are either unaffected (POM-1) or drastically reduced (SMB-1) when these enzymes are expressed in *E. coli* MC1000 *dsbA*. Protein levels of StrepII-tagged β-lactamases were assessed using a Strep-Tactin-AP conjugate. A representative blot from three biological experiments, each conducted as a single technical repeat, is shown; molecular weight markers (M) are on the left, DnaK was used as a loading control and solid black lines indicate where the membrane was cut. Full immunoblots and SDS PAGE analysis of the immunoblot samples for total protein content are shown in File S3. **(C)** The hydrolytic activities of both tested β-lactamases are significantly reduced in the absence of DsbA. The hydrolytic activities of strains harboring the empty vector or expressing the control enzyme L2-1 show no dependence on DsbA; the same data for the control strains are also shown in Fig. 2B. n=3 (each conducted in technical duplicate), table shows means ± SD, significance is indicated by * = $p < 0.05$, ns = non-significant.

bioRxiv preprint doi: https://doi.org/10.1101/2023.08.02.551661; this version posted June 2, 2025. The copyright holder for this preprint (which was not certified by peer review) is the author/funder, who has granted bioRxiv a license to display the preprint in perpetuity. It is made available under aCC-BY 4.0 International license.

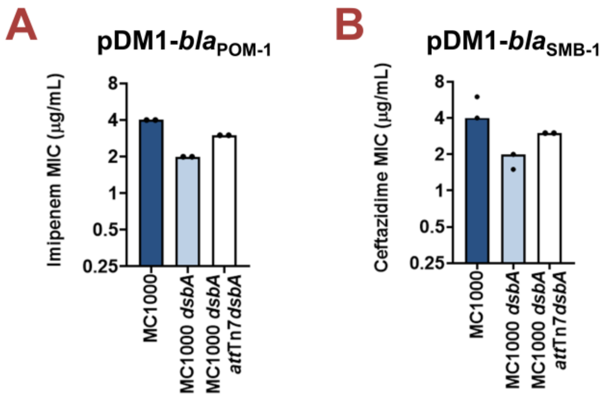


**Figure S5. Complementation of *dsbA* restores the β-lactam MIC values for *E. coli* MC1000 *dsbA* expressing β-lactamases.** Re-insertion of *dsbA* at the *att*Tn*7* site of the chromosome restores representative β-lactam MIC values for *E. coli* MC1000 *dsbA* harboring **(A)** pDM1-*bla*$_{\text{POM-1}}$ (imipenem MIC), and **(B)** pDM1-*bla*$_{\text{SMB-1}}$ (ceftazidime MIC). Graphs show MIC values (µg/mL) and are representative of two biological experiments, each conducted as a single technical repeat.

bioRxiv preprint doi: https://doi.org/10.1101/2023.08.02.551661; this version posted June 2, 2025. The copyright holder for this preprint (which was not certified by peer review) is the author/funder, who has granted bioRxiv a license to display the preprint in perpetuity. It is made available under aCC-BY 4.0 International license.

## SUPPLEMENTARY TABLES

**Table S1.** Overview of the β-lactamase enzymes investigated in this study. All tested enzymes belong to distinct phylogenetic clusters (see File S1), with the exception of BPS-1m and BPS-6. The "Cysteine positions" column states the positions of cysteine residues after amino acid 30 and hence, does not include amino acids that are part of the periplasmic signal sequence which is cleaved after protein translocation. All β-lactamase enzymes except L2-1 and LUT-1 (shaded in grey), which are used as negative controls throughout this study, have one or more disulfide bonds. Both L2-1 and LUT-1 contain two or more cysteine residues, but lack disulfide bonds as they are transported to the periplasm in a folded state by the Twin-arginine translocation (Tat) system; for L2-1 Tat-dependent translocation has been experimentally confirmed [2], whereas for LUT-1 this is strongly corroborated by signal peptide prediction software (SignalP 5.0 [3] likelihood scores: Sec/SPI = 0.0572, Tat/SPI = 0.9312, Sec/SPII (lipoprotein) = 0.0087, other = 0.0029). The "Mob." (mobilizable) column refers to the possibility for the β-lactamase gene to be mobilized from the chromosome; "yes" indicates that the gene of interest is located on a mobile element, while "no" refers to immobile chromosomally-encoded enzymes. The "Spectrum" column refers to the hydrolytic spectrum of each tested enzyme; tested enzymes are narrow-spectrum β-lactamases (NS), extended-spectrum β-lactamases (ESBL) or carbapenemases. The "Inh." (inhibition) column refers to classical inhibitor susceptibility i.e., susceptibility to inhibition by clavulanic acid, tazobactam or sulbactam. Finally, the "Organism" column refers to the bacterial species that most commonly express the tested β-lactamase enzymes.

| ENZYME | CYSTEINE POSITIONS | AMBLER CLASS | MOB. | SPECTRUM | INH. | ORGANISM |
|---|---|---|---|---|---|---|
| **L2-1** | C82 C136 C233 | A | no | ESBL | yes | *Stenotrophomonas maltophilia* |
| **LUT-1** | C54 C129 | A | no [4] | NS | yes | *Pseudomonas luteola* |
| **BEL-1** | C61 C231 | A | yes [5] | ESBL | yes | *Pseudomonas aeruginosa* |
| **CARB-2** | C72 C118 | A | yes [6] | NS | yes | *Pseudomonas spp.* |
| **BPS-1m** | C75 C83 C129 | A | no [7] | ESBL | yes | *Burkholderia pseudomallei* |
| **BPS-6** | C75 C83 C129 | A | no [8] | ESBL | yes | *Burkholderia pseudomallei* |
| **GES-19, 20, or 26** | C63 C233 | A | yes [9] | ESBLs | yes | *Enterobacteriaceae, Pseudomonas aeruginosa* |
| **AIM-1** | C31 C56 C194 C199 C234 C274 | B3 | yes [10] | carbapenemase | no [11] | *Pseudomonas aeruginosa* |
| **L1-1** | C239 C265 | B3 | no [11] | carbapenemase | no [11] | *Stenotrophomonas maltophilia* |
| **POM-1** | C237 C265 | B3 | no [12] | carbapenemase | no [11] | *Pseudomonas otitidis* |
| **SMB-1** | C180 C 185 C226 C260 | B3 | yes [13] | carbapenemase | no [11] | *Serratia spp.* |
| **OXA-50** | C208 C211 | D | no [14] | NS | no [14] | *Pseudomonas spp.* |

bioRxiv preprint doi: https://doi.org/10.1101/2023.08.02.551661; this version posted June 2, 2025. The copyright holder for this preprint (which was not certified by peer review) is the author/funder, who has granted bioRxiv a license to display the preprint in perpetuity. It is made available under aCC-BY 4.0 International license.

**Table S2.** Bacterial strains used in this study. All listed isolates are clinical strains. "FNRCAR" refers to the French National Reference Centre for Antibiotic Resistance in Le Kremlin-Bicêtre, France, and "CDC AR Isolate bank" refers to the Centers for Disease Control and Prevention Antibiotic Resistance Isolate Bank in Atlanta, GA, USA.

| NAME | DESCRIPTION | SOURCE |
|---|---|---|
| ***Escherichia coli*** | | |
| DH5α | F⁻ *end*A1 *gln*V44 *thi*-1 *rec*A1 *rel*A1 *gyr*A96 *deo*R *nup*G *pur*B20 φ80d*lac*ZΔM15 Δ(*lac*ZYA-*arg*F)U169 *hsd*R17($r_K^-m_K^+$) λ⁻ | [15] |
| DH5αλpir | λ*pir* | [16] |
| CC118λpir | *ara*D Δ(*ara*, *leu*) Δ*lac*Z74 *pho*A20 *gal*K *thi*-1 *rsp*E *rpo*B *arg*E *rec*A1 λ*pir* | [17] |
| HB101 | *sup*E44 *hsd*S20 *rec*A13 *ara*-14 *pro*A2 *lac*Y1 *gal*K2 *rps*L20 *xyl*-5 *mtl*-1 | [18] |
| MC1000 | *ara*D139 Δ(*ara*, *leu*)7697 Δ*lac*X74 *gal*U *gal*K *str*A | [19] |
| MC1000 *dsbA* | *dsbA*::*aphA*, $Kan^R$ | [20] |
| MC1000 *dsbA att*Tn*7*::P*tac*-*dsbA* | *dsbA*::*aphA att*Tn*7*::*dsbA*, $Kan^R$ | [1] |
| **Clinical isolates** | | |
| *Pseudomonas aeruginosa* PAO1 | wild-type prototroph | [21] |
| *Pseudomonas aeruginosa* PA14 | wild-type prototroph | [22] |
| *Pseudomonas aeruginosa* PA14 *att*Tn*7*::*accC* | *att*Tn*7*::*accC*, $Gent^R$ | This study |
| *Pseudomonas aeruginosa* G4R7 | $bla_{AIM-1}$ | FNRCAR |
| *Pseudomonas aeruginosa* G4R7 *dsbA1* | *dsbA1* $bla_{AIM-1}$ | This study |
| *Pseudomonas aeruginosa* G6R7 | $bla_{AIM-1}$ | FNRCAR |
| *Pseudomonas aeruginosa* G6R7 *dsbA1* | *dsbA1* $bla_{AIM-1}$ | This study |
| *Pseudomonas aeruginosa* CDC #769 | $bla_{GES-19}$ $bla_{GES-26}$ | CDC AR Isolate Bank |
| *Pseudomonas aeruginosa* CDC #769 *dsbA1* | *dsbA1* $bla_{GES-19}$ $bla_{GES-26}$ | This study |
| *Pseudomonas aeruginosa* CDC #769 *dsbA1 att*Tn*7*::*accC msfgfp dsbA1* | *dsbA1* $bla_{GES-19}$ $bla_{GES-26}$ *att*Tn*7*::*accC msfgfp dsbA1*, $Gent^R$ | This study |
| *Pseudomonas aeruginosa* CDC #773 | $bla_{GES-19}$ $bla_{GES-20}$ | CDC AR Isolate Bank |
| *Pseudomonas aeruginosa* CDC #773 *dsbA1* | *dsbA1* $bla_{GES-19}$ $bla_{GES-20}$ | This study |
| *Stenotrophomonas maltophilia* AMM | $bla_{L2-1}$ $bla_{L1-1}$ | [23] |
| *Stenotrophomonas maltophilia* AMM *dsbA dsbL* | *dsbA dsbL* $bla_{L2-1}$ $bla_{L1-1}$ | This study |
| *Stenotrophomonas maltophilia* AMM | $bla_{L2-1}$ $bla_{L1-1}$ | This study |

bioRxiv preprint doi: https://doi.org/10.1101/2023.08.02.551661; this version posted June 2, 2025. The copyright holder for this preprint (which was not certified by peer review) is the author/funder, who has granted bioRxiv a license to display the preprint in perpetuity. It is made available under aCC-BY 4.0 International license.

| | | |
|---|---|---|
| *att*Tn*7::accC msfgfp* | *att*Tn*7::accC msfgfp*, Gent$^R$ | |
| *Stenotrophomonas maltophilia* AMM *dsbA dsbL att*Tn*7::accC msfgfp* | *dsbA dsbL* $bla_{L2\text{-}1}$ $bla_{L1\text{-}1}$ *att*Tn*7::accC msfgfp*, Gent$^R$ | This study |
| *Stenotrophomonas maltophilia* AMM *dsbA dsbL att*Tn7*::accC msfgfp dsbA1* | *dsbA dsbL* $bla_{L2\text{-}1}$ $bla_{L1\text{-}1}$ *att*Tn*7::accC msfgfp dsbA1*, Gent$^R$ | This study |
| *Stenotrophomonas maltophilia* GUE | $bla_{L2\text{-}1}$ $bla_{L1\text{-}1}$ | [23] |
| *Stenotrophomonas maltophilia* GUE *dsbA dsbL* | *dsbA dsbL* $bla_{L2\text{-}1}$ $bla_{L1\text{-}1}$ | This study |

bioRxiv preprint doi: https://doi.org/10.1101/2023.08.02.551661; this version posted June 2, 2025. The copyright holder for this preprint (which was not certified by peer review) is the author/funder, who has granted bioRxiv a license to display the preprint in perpetuity. It is made available under aCC-BY 4.0 International license.

**Table S3.** Plasmids used in this study.

| NAME | DESCRIPTION | SOURCE |
|---|---|---|
| pDM1 | pDM1 vector (GenBank MN128719), p15A *ori*, P*tac* promoter, MCS, $Tet^R$ | Mavridou lab stock |
| pDM1-*bla*$_{L2\text{-}1}$ | *bla*$_{L2\text{-}1}$ cloned into pDM1, $Tet^R$ | [1] |
| pDM1-*bla*$_{LUT\text{-}1}$ | *bla*$_{LUT\text{-}1}$ cloned into pDM1, $Tet^R$ | This study |
| pDM1-*bla*$_{BEL\text{-}1}$ | *bla*$_{BEL\text{-}1}$ cloned into pDM1, $Tet^R$ | This study |
| pDM1-*bla*$_{CARB\text{-}2}$ | *bla*$_{CARB\text{-}2}$ cloned into pDM1, $Tet^R$ | This study |
| pDM1-*bla*$_{BPS\text{-}1m}$ | *bla*$_{BPS\text{-}1m}$ cloned into pDM1, $Tet^R$ | This study |
| pDM1-*bla*$_{BPS\text{-}6}$ | *bla*$_{BPS\text{-}6}$ cloned into pDM1, $Tet^R$ | This study |
| pDM1-*bla*$_{AIM\text{-}1}$ | *bla*$_{AIM\text{-}1}$ cloned into pDM1, $Tet^R$ | This study |
| pDM1-*bla*$_{POM\text{-}1}$ | *bla*$_{POM\text{-}1}$ cloned into pDM1, $Tet^R$ | This study |
| pDM1-*bla*$_{SMB\text{-}1}$ | *bla*$_{SMB\text{-}1}$ cloned into pDM1, $Tet^R$ | This study |
| pDM1-*bla*$_{OXA\text{-}50}$ | *bla*$_{OXA\text{-}50}$ cloned into pDM1, $Tet^R$ | This study |
| pDM1-*bla*$_{BEL\text{-}1}$-StrepII | *bla*$_{BEL\text{-}1}$ encoding BEL-1 with a C-terminal StrepII tag cloned into pDM1, $Tet^R$ | This study |
| pDM1-*bla*$_{CARB\text{-}2}$-StrepII | *bla*$_{CARB\text{-}2}$ encoding CARB-2 with a C-terminal StrepII tag cloned into pDM1, $Tet^R$ | This study |
| pDM1-*bla*$_{BPS\text{-}1m}$-StrepII | *bla*$_{BPS\text{-}1m}$ encoding BPS-1m with a C-terminal StrepII tag cloned into pDM1, $Tet^R$ | This study |
| pDM1-*bla*$_{AIM\text{-}1}$-StrepII | *bla*$_{AIM\text{-}1}$ encoding AIM-1 with a C-terminal StrepII tag cloned into pDM1, $Tet^R$ | This study |
| pDM1-*bla*$_{L1\text{-}1}$-StrepII | *bla*$_{L1\text{-}1}$ encoding L1-1 with a C-terminal StrepII tag cloned into pDM1, $Tet^R$ | [1] |
| pDM1-*bla*$_{POM\text{-}1}$-StrepII | *bla*$_{POM\text{-}1}$ encoding POM-1 with a C-terminal StrepII tag cloned into pDM1, $Tet^R$ | This study |
| pDM1-*bla*$_{SMB\text{-}1}$-StrepII | *bla*$_{SMB\text{-}1}$ encoding SMB-1 with a C-terminal StrepII tag cloned into pDM1, $Tet^R$ | This study |
| pDM1-*bla*$_{OXA\text{-}50}$-StrepII | *bla*$_{OXA\text{-}50}$ encoding OXA-50 with a C-terminal StrepII tag cloned into pDM1, $Tet^R$ | This study |
| pKNG101 | Gene replacement suicide vector, *ori*R6K, *ori*TRK2, *sacB*, $Str^R$ | [24] |
| pKNG102 | Gene replacement suicide vector, *ori*R6K, *ori*TRK2, *sacB*, $Tet^R$ | Bernal lab stock |
| pKNG101-*dsbA1* | PCR fragment containing the regions upstream and downstream *P. aeruginosa dsbA1* cloned in pKNG101; when inserted into the chromosome, the strain is a merodiploid for *dsbA1* mutant, $Str^R$ | [1] |
| pKNG102-*dsbA1*-769 | PCR fragment containing the regions upstream and downstream *P. aeruginosa* CDC #769 (Table S2) *dsbA1* cloned in pKNG102; when inserted into the chromosome, the strain is a merodiploid for *dsbA1* mutant, $Tet^R$ | This study |
| pKNG102-*dsbA1*-773 | PCR fragment containing the regions upstream and downstream *P. aeruginosa* CDC #773 (Table S2) *dsbA1* cloned in pKNG102; when inserted into the | This study |

bioRxiv preprint doi: https://doi.org/10.1101/2023.08.02.551661; this version posted June 2, 2025. The copyright holder for this preprint (which was not certified by peer review) is the author/funder, who has granted bioRxiv a license to display the preprint in perpetuity. It is made available under aCC-BY 4.0 International license.

| | | |
|---|---|---|
| | chromosome, the strain is a merodiploid for *dsbA1* mutant, $Tet^R$ | |
| pKNG101-*dsbA dsbL*-AMM | PCR fragment containing the regions upstream and downstream *S. maltophilia* AMM *dsbA* and *dsbL* genes cloned in pKNG101; when inserted into the chromosome, the strain is a merodiploid for *dsbA dsbL* mutant, $Str^R$ | This study |
| pKNG101-*dsbA dsbL*-GUE | PCR fragment containing the regions upstream and downstream *S. maltophilia* GUE *dsbA* and *dsbL* genes cloned in pKNG101; when inserted into the chromosome, the strain is a merodiploid for *dsbA dsbL* mutant, $Str^R$ | This study |
| pRK600 | Helper plasmid, ColE1 *ori*, *mob*RK2, *tra*RK2, $Cam^R$ | [25] |
| pTn*7*-M | Mini-Tn*7* delivery transposon vector containing the Tn*7* flanking regions and a $Gent^R$ marker, R6K *ori*, $Kan^R$, $Gent^R$ | [26] |
| pBG42 | Mini-Tn*7* delivery transposon vector containing the Tn*7* flanking regions, a $Gent^R$ marker and *msfgfp*, R6K *ori*, $Kan^R$, $Gent^R$ | [26] |
| pBG42-PAO1*dsbA1* | *dsbA1* encoding DsbA1 from *P. aeruginosa* PAO1 cloned into pBG42, $Kan^R$, $Gent^R$ | This study |
| pTNS2 | Helper plasmid, R6K *ori*; encodes the TnsABC+D specific transposition pathway, $Amp^R$ | [27] |
| pMK-RQ *carb-2* | GeneArt® cloning vector containing *carb-2*, ColE1 *ori*, (template for *carb-2*), $Kan^R$ | This study |
| pMK-RQ *bps-1m* | GeneArt® cloning vector containing *bps-1m*, ColE1 *ori*, (template for *bps-1m*), $Kan^R$ | This study |
| pMK-RQ *bps-6* | GeneArt® cloning vector containing *bps-6*, ColE1 *ori*, (template for *bps-6*), $Kan^R$ | This study |
| pMK-RQ *smb-1* | GeneArt® cloning vector containing *smb-1*, ColE1 *ori*, (template for *smb-1*), $Kan^R$ | This study |

bioRxiv preprint doi: https://doi.org/10.1101/2023.08.02.551661; this version posted June 2, 2025. The copyright holder for this preprint (which was not certified by peer review) is the author/funder, who has granted bioRxiv a license to display the preprint in perpetuity. It is made available under aCC-BY 4.0 International license.

**Table S4.** Oligonucleotide primers used in this study. The "Brief description" column provides basic information on the primer design (restriction enzyme used for cloning, encoded protein or gene replaced by antibiotic resistance cassette, forward or reverse orientation of the primer (F or R); SQ stands for sequencing primers).

| NUMBER | BRIEF DESCRIPTION | SEQUENCE (5´-3´) |
|---|---|---|
| P1 | SacI.LUT-1.F | ctggagctcaatgtcatcctgaaccgtcga |
| P2 | PstI.LUT-1.R | cagctgcagtcagcctgtcacccattcag |
| P3 | SacI.BEL-1.F | ctggagctcaaactgctctacccgttattgc |
| P4 | PstI.BEL-1.R | cagctgcagtcagtgaacatattgacgtgc |
| P5 | SacI.CARB-2.F | ctggagctcaagtttttattggcattttcgc |
| P6 | KpnI.CARB-2.R | cagggtacctcagcgcgactgtgatgta |
| P7 | SacI.BPS-1m.F | ctggagctcaatcattctccgttgcgccgctc |
| P8 | XmaI.BPS-1m.R | caacccgggtcaggcgaacgcccgcgcg |
| P9 | SacI.AIM-1.F | ctggagctcaaacgtcgcttcaccctgg |
| P10 | KpnI.AIM-1.R | ctgggtacctcaaggccgcgcgccgctg |
| P11 | SacI.POM-1.F | ctggagctccgtaccctgaccctcg |
| P12 | KpnI.POM-1.R | cagggtaccttatgcgtcatcagagacctc |
| P13 | NdeI.SMB-1.F | cagctccatatgaaaatcatcgcttccctgatcc |
| P14 | XmaI.SMB-1.R | ctgcccgggtcagcgtttctcgctggcca |
| P15 | SacI.OXA-50.F | ctggagctccgccctctcttcagtg |
| P16 | KpnI.OXA-50.R | cagggtacctcagggcagtatcccgagag |
| P17 | PstI.StrepII.BEL-1.R | cagctgcagttatttttcaaattgcggatggctccaagcgctc ccgtgaacatattgacgtgctaac |
| P18 | KpnI.StrepII.CARB-2.R | cagggtaccttatttttcaaattgcggatggctccaagcgctc ccgcgcgactgtgatgtataa |
| P19 | XmaI.StrepII.BPS-1m.R | ctgcccgggctatttttcaaattgcggatggctccaagcgct cccggcgaacgcccgcgcggcg |
| P20 | KpnI.StrepII.AIM-1.R | cagggtaccttatttttcaaattgcggatggctccaagcgctc ccaggccgcgcgccgctggag |
| P21 | KpnI.StrepII.POM-1.R | cagggtaccttatttttcaaattgcggatggctccaagcgctc ccgccgcgctgcttc |
| P22 | XmaI.StrepII.SMB-1.R | ctgcccgggctatttttcaaattgcggatggctccaagcgct cccgcgtttctcgctggccag |
| P23 | KpnI.StrepII.OXA-50.R | cagggtaccttatttttcaaattgcggatggctccaagcgctc ccgggcagtatcccgagagcc |
| P24 | SQ.dsbA1.Paeruginosa.F | tacctgctcaagcagatgcatg |
| P25 | SQ.dsbA1.Paeruginosa.R | ggtgttcatgtcgcccatca |
| P26 | SQ.dsbAdsbL.Smaltophilia.F | atggtgccgttcgtgcaga |
| P27 | SQ.dsbAdsbL.Smaltophilia.R | acagcacctgcatttccgg |
| P28 | XbaI.dsbA1.F | ggttcctctagagcctacttcgccagccagaa |
| P29 | pKNG101-dsbA1.body.R | ctacttcttgttacgcatcgttcactc |
| P30 | pKNG101-dsbA1.body.F | atgcgtaacaagaagtaggcaaggtga |
| P31 | BamHI.dsbA1.R | aattaaggatcctcatcactaccaccagcgcg |
| P32 | XbaI.dsbAdsbL.F | ggttcctctagatcttctggtacagcacctgcatttccg |
| P33 | pKNG102-dsbAdsbL.body.R | tgcgtgtcgatgaggttggctcactga |
| P34 | pKNG102-dsbAdsbL.body.F | tctcttggatcagtgagccaacctcat |
| P35 | BamHI.dsbAdsbL.R | aattaaggatcctcgctggaggtggatttcagcaagacc |
| P36 | pBG42-vector.F | gaattcgagctcggtaccc |
| P37 | pBG42-vector.R | tagaaaacctccttagcatgattaagatg |

bioRxiv preprint doi: https://doi.org/10.1101/2023.08.02.551661; this version posted June 2, 2025. The copyright holder for this preprint (which was not certified by peer review) is the author/funder, who has granted bioRxiv a license to display the preprint in perpetuity. It is made available under aCC-BY 4.0 International license.

| | | |
|---|---|---|
| P38 | PAO1dsbA1-insert.F | catgctaaggaggttttctaatgcgtaacctgattctcacc |
| P39 | PAO1dsbA1-insert.R | gtaccgagctcgaattcctacttcttggccgctgc |
| P40 | HindIII.PEM7-msfgfp.F | cacaaagctttgttgacaattaatcatcggcatagtatatcggcatagtataatacgacaaggtgaggaactaaaccaggaggaaaaacatatgcgtaaaggtgaagaactgttcac |
| P41 | msfgfp.BamHI.R | cacaggatccttatttgtagagttcatccatgccg |
| P42 | SQ.pBG42-PAO1dsbA1.F | ccgctgcgttcggtc |
| P43 | SQ.pBG42-PAO1dsbA1.R | ccaagactagtcgccagg |
| P44 | SQ.Tn7.Paeruginosa.F | gtcgaagccgagctggtg |
| P45 | SQ.Tn7.Paeruginosa.R | gatcgccaagggtgcctg |
| P46 | SQ.Tn7.Smaltophilia.F | gtcgatgccgcccaagaag |
| P47 | SQ.Tn7.Smaltophilia.R | gatggcaccttccatgagaac |

bioRxiv preprint doi: https://doi.org/10.1101/2023.08.02.551661; this version posted June 2, 2025. The copyright holder for this preprint (which was not certified by peer review) is the author/funder, who has granted bioRxiv a license to display the preprint in perpetuity. It is made available under aCC-BY 4.0 International license.

**Table S5.** Sources of genomic DNA used for amplification of β-lactamase genes used in this study. CRBIP stands for Centre de Ressources Biologiques de l'Institut Pasteur, France and FNRCAR refers to the French National Reference Centre for Antibiotic Resistance in Le Kremlin-Bicêtre, France.

| STRAIN | GENE | SOURCE |
|---|---|---|
| *Pseudomonas aeruginosa* 51170 | *bla*$_{\text{BEL-1}}$ | [5] |
| *Pseudomonas luteola* CIP 102067 | *bla*$_{\text{LUT-1}}$ | CRBIP |
| *Pseudomonas aeruginosa* G4R7 | *bla*$_{\text{AIM-1}}$ | FNRCAR |
| *Pseudomonas otitidis* CIP 109236T | *bla*$_{\text{POM-1}}$ | CRBIP |
| *Pseudomonas aeruginosa* PAO1 LA | *bla*$_{\text{OXA-50}}$ | [21] |

bioRxiv preprint doi: https://doi.org/10.1101/2023.08.02.551661; this version posted June 2, 2025. The copyright holder for this preprint (which was not certified by peer review) is the author/funder, who has granted bioRxiv a license to display the preprint in perpetuity. It is made available under aCC-BY 4.0 International license.

## LEGENDS FOR SUPPLEMENTARY DATA FILES

**File S1. Analysis of the cysteine content and phylogeny of all identified β-lactamases.** 7,741 unique β-lactamase protein sequences were clustered with a 90% identity threshold and the centroid of each cluster was used as a phylogenetic cluster identifier for each sequence ("Phylogenetic cluster (90% ID)" column). All sequences were searched for the presence of cysteine residues ("Total number of cysteines" and "Positions of all cysteines" columns). Proteins with two or more cysteines after the first 30 amino acids of their primary sequence (cells shaded in grey in the "Number of cysteines after position 30" column) are potential substrates of the DSB system for organisms where oxidative protein folding is carried out by DsbA and provided that translocation of the β-lactamase outside the cytoplasm is performed by the Sec system. The first 30 amino acids of each sequence were excluded to avoid considering cysteines that are part of the signal sequence mediating the translocation of these enzymes outside the cytoplasm. Cells shaded in grey in the "Reported in pathogens" column mark β-lactamases that are found in pathogens or organisms capable of causing opportunistic infections. The Ambler class of each enzyme is indicated in the "Ambler class column" and each class (A, B1, B2, B3, C and D) is highlighted in a different color.

**File S2. Data used to generate Fig. 1, Fig. S4, Fig. 4B and Fig. S3B. (A)** MIC values (µg/mL) used to generate Fig. 1 are in rows 2-7 [strains serving as negative controls; *E. coli* MC1000 strains harboring pDM1 (vector alone), pDM1-*bla*$_{L2\text{-}1}$ or pDM1-*bla*$_{LUT\text{-}1}$ (cysteine-containing β-lactamases which lack disulfide bonds)] and rows 9-20. MIC values (µg/mL) used to generate Fig. S4 are in rows 22-25. The aminoglycoside antibiotic gentamicin serves as a negative control for all strains. Cells marked with a dash (-) represent strain-antibiotic combinations that were not tested. **(B)** *P. aeruginosa* PA14 colony forming unit (CFU) counts used to generate Fig. 4E. **(C)** *P. aeruginosa* PA14, *S. maltophilia* AMM and *S. maltophilia AMM dsbA dsbL* CFU counts used to generate Fig. S3B. For all tabs, three biological experiments are shown; for (B) and (C) each biological replicate was conducted in technical triplicate and mean CFU values are shown.

**File S3. Full immunoblots and SDS PAGE analysis of the immunoblot samples for total protein content.** (**Pages 1-6**) Full immunoblots for Fig. 2A and S4B. On the left of each page, the relevant figure panel is shown and the lanes in question are marked with red outline. On the right of each page, the full immunoblot is displayed with the corresponding area also marked with red outline. (**Pages 7-9**) SDS PAGE analysis of the immunoblot samples for total protein content. In each page, the immunoblot in question is indicated (by "Fig. 2A" or "Fig. S4B") and lanes are marked accordingly to identify the immunoblot lane that they correspond to (see white labels at the bottom of the gel).

**File S4. Analysis of *Stenotrophomonas spp.* for the presence of MCR proteins.** Hidden Markov Models built from validated sequences of MCR-like and EptA-like proteins were used for the identification of MCR-like analogues in a total of 106 complete genomes of the *Stenotrophomonas* genus downloaded from the NCBI repository. **(A)** Most genomes that were investigated ("*Stenotrophomonas maltophilia* genome" column"), encoded one or two MCR-like proteins ("Number of MCR analogues column"). **(B)** The 146 MCR-like sequences ("Protein ID column") that were identified (only hits with evalues < 1e-10 were considered; "Evalue" column) belong to the same phylogenetic group as validated MCR-5 or MCR-8 proteins ("Phylogenetic group" column).

bioRxiv preprint doi: https://doi.org/10.1101/2023.08.02.551661; this version posted June 2, 2025. The copyright holder for this preprint (which was not certified by peer review) is the author/funder, who has granted bioRxiv a license to display the preprint in perpetuity. It is made available under aCC-BY 4.0 International license.

**File S5. Quality control information on 4,5-dibromo-2-(2-chlorobenzyl)pyridazin-3(2H)-one.** $^{1}$H-NMR and LCMS spectra of 4,5-dibromo-2-(2-chlorobenzyl)pyridazin-3(2H)-one (compound 36) demonstrating the correctness and purity of the synthesized compound by Bioduro-Sundia.

bioRxiv preprint doi: https://doi.org/10.1101/2023.08.02.551661; this version posted June 2, 2025. The copyright holder for this preprint (which was not certified by peer review) is the author/funder, who has granted bioRxiv a license to display the preprint in perpetuity. It is made available under aCC-BY 4.0 International license.

## SUPPLEMENTARY REFERENCES


1. Furniss RCD, Kaderabkova N, Barker D, Bernal P, Maslova E, et al. (2022) Breaking antimicrobial resistance by disrupting extracytoplasmic protein folding. eLife 11: e57974.

2. Pradel N, Delmas J, Wu LF, Santini CL, Bonnet R (2009) Sec- and Tat-dependent translocation of b-lactamases across the *Escherichia coli* inner membrane. Antimicrob. Agents Chemother. 53: 242-248.

3. Almagro Armenteros JJ, Tsirigos KD, Sonderby CK, Petersen TN, Winther O, et al. (2019) SignalP 5.0 improves signal peptide predictions using deep neural networks. Nat. Biotechnol. 37: 420-423.

4. Doublet B, Robin F, Casin I, Fabre L, Le Fleche A, et al. (2010) Molecular and biochemical characterization of the natural chromosome-encoded class A β-lactamase from *Pseudomonas luteola*. Antimicrob. Agents Chemother. 54: 45-51.

5. Poirel L, Brinas L, Verlinde A, Ide L, Nordmann P (2005) BEL-1, a novel clavulanic acid-inhibited extended-spectrum β-lactamase, and the class 1 integron In120 in *Pseudomonas aeruginosa*. Antimicrob. Agents Chemother. 49: 3743-3748.

6. Bert F, Branger C, Lambert-Zechovsky N (2002) Identification of PSE and OXA b-lactamase genes in *Pseudomonas aeruginosa* using PCR-restriction fragment length polymorphism. J. Antimicrob. Chemother. 50: 11-18.

7. Ho PL, Cheung TKM, Yam WC, Yuen KY (2002) Characterization of a laboratory-generated variant of BPS b-lactamase from *Burkholderia pseudomallei* that hydrolyses ceftazidime. J. Antimicrob. Chemother. 50: 723-726.

8. Tribuddharat C, Moore RA, Baker P, Woods DE (2003) *Burkholderia pseudomallei* class a b-lactamase mutations that confer selective resistance against ceftazidime or clavulanic acid inhibition. Antimicrob. Agents Chemother. 47: 2082-2087.

9. Yoon EJ, Jeong SH (2021) Mobile carbapenemase genes in *Pseudomonas aeruginosa*. Front. Microbiol. 12: 614058.

10. Yong D, Toleman MA, Bell J, Ritchie B, Pratt R, et al. (2012) Genetic and biochemical characterization of an acquired subgroup B3 metallo-β-lactamase gene, $bla_{\text{AIM-1}}$, and its unique genetic context in *Pseudomonas aeruginosa* from Australia. Antimicrob. Agents Chemother. 56: 6154-6159.

11. Tooke CL, Hinchliffe P, Bragginton EC, Colenso CK, Hirvonen VHA, et al. (2019) β-Lactamases and β-lactamase inhibitors in the 21st century. J. Mol. Biol. 431: 3472-3500.

12. Thaller MC, Borgianni L, Di Lallo G, Chong Y, Lee K, et al. (2011) Metallo-β-lactamase production by *Pseudomonas otitidis*: a species-related trait. Antimicrob. Agents Chemother. 55: 118-123.

13. Wachino J, Yoshida H, Yamane K, Suzuki S, Matsui M, et al. (2011) SMB-1, a novel subclass B3 metallo-β-lactamase, associated with ISCR1 and a class 1 integron, from a carbapenem-resistant *Serratia marcescens* clinical isolate. Antimicrob. Agents Chemother. 55: 5143-5149.

14. Girlich D, Naas T, Nordmann P (2004) Biochemical characterization of the naturally occurring oxacillinase OXA-50 of *Pseudomonas aeruginosa*. Antimicrob. Agents Chemother. 48: 2043-2048.

bioRxiv preprint doi: https://doi.org/10.1101/2023.08.02.551661; this version posted June 2, 2025. The copyright holder for this preprint (which was not certified by peer review) is the author/funder, who has granted bioRxiv a license to display the preprint in perpetuity. It is made available under aCC-BY 4.0 International license.

15. Hanahan D (1985) In: Glover DM and Hames BD, editors. DNA cloning: a practical approach: IRL Press, McLean, Virginia. pp. 109.

16. Martinez-Garcia E, de Lorenzo V (2011) Engineering multiple genomic deletions in Gram-negative bacteria: analysis of the multi-resistant antibiotic profile of *Pseudomonas putida* KT2440. Environ. Microbiol. 13: 2702-2716.

17. Herrero M, de Lorenzo V, Timmis KN (1990) Transposon vectors containing non-antibiotic resistance selection markers for cloning and stable chromosomal insertion of foreign genes in Gram-negative bacteria. J. Bacteriol. 172: 6557-6567.

18. Boyer HW, Roulland-Dussoix D (1969) A complementation analysis of the restriction and modification of DNA in *Escherichia coli*. J. Mol. Biol. 41: 459-472.

19. Casadaban MJ, Cohen SN (1980) Analysis of gene control signals by DNA fusion and cloning in *Escherichia coli*. J. Mol. Biol. 138: 179-207.

20. Kadokura H, Tian H, Zander T, Bardwell JC, Beckwith J (2004) Snapshots of DsbA in action: detection of proteins in the process of oxidative folding. Science 303: 534-537.

21. Holloway BW (1969) Genetics of *Pseudomonas*. Bacteriol. Rev. 33: 419-443.

22. Rahme LG, Stevens EJ, Wolfort SF, Shao J, Tompkins RG, et al. (1995) Common virulence factors for bacterial pathogenicity in plants and animals. Science 268: 1899-1902.

23. Emeraud C, Escaut L, Boucly A, Fortineau N, Bonnin RA, et al. (2019) Aztreonam plus clavulanate, tazobactam, or avibactam for treatment of infections caused by metallo-β-lactamase-producing Gram-negative bacteria. Antimicrob. Agents Chemother. 63.

24. Kaniga K, Delor I, Cornelis GR (1991) A wide-host-range suicide vector for improving reverse genetics in Gram-negative bacteria: inactivation of the *blaA* gene of *Yersinia enterocolitica*. Gene 109: 137-141.

25. Kessler B, Delorenzo V, Timmis KN (1992) A general system to integrate *lacZ* fusions into the chromosomes of Gram-negative eubacteria: regulation of the *Pm* Promoter of the *TOL* plasmid studied with all controlling elements in monocopy. Mol. Gen. Genet. 233: 293-301.

26. Zobel S, Benedetti I, Eisenbach L, de Lorenzo V, Wierckx N, et al. (2015) Tn*7*-based device for calibrated heterologous gene expression in *Pseudomonas putida*. ACS Synth. Biol. 4: 1341-1351.

27. Choi KH, Gaynor JB, White KG, Lopez C, Bosio CM, et al. (2005) A Tn*7*-based broad-range bacterial cloning and expression system. Nat. Methods 2: 443-448.
