# Supplementary material for: Antibiotic potentiation and inhibition of cross-resistance in pathogens associated with cystic fibrosis
Source: bioRxiv. 2023 Aug 2:2023.08.02.551661. Preprint. [Version 1] doi: 10.1101/2023.08.02.551661 (PMC10418187; doi:10.1101/2023.08.02.551661)
Supplement: Supplementary file 1 [file media-1.pdf]

SUPPLEMENTARY INFORMATION FOR

Antibiotic potentiation and inhibition of cross-resistance in pathogens associated with cystic fibrosis

Nikol Kadeřábková[1,3], R. Christopher D. Furniss[3], Evgenia Maslova[4], Lara Eisaiankhongi[4], Patricia Bernal[5], Alain Filloux[3,6], Cristina Landeta[7], Diego Gonzalez[8], Ronan R. McCarthy[4], Despoina A.I. Mavridou[1,2*]

[1]Department of Molecular Biosciences, The University of Texas at Austin, Austin, 78712, Texas, USA
[2]John Ring LaMontagne Center for Infectious Diseases, The University of Texas at Austin, Austin, 78712, Texas, USA
[3]Centre for Bacterial Resistance Biology, Department of Life Sciences, Imperial College London, London, SW7 2AZ, UK
[4]Division of Biosciences, Department of Life Sciences, College of Health and Life Sciences, Brunel University London, Uxbridge, UB8 3PH, UK
[5]Departamento de Microbiología, Facultad de Biología, Universidad de Sevilla, Seville, 41012, Spain
[6]Singapore Centre for Environmental Life Sciences Engineering, Nanyang Technological University, 637551, Singapore
[7]Department of Biology, Indiana University, Bloomington, Indiana, 47405, USA.
[8]Laboratoire de Microbiologie, Institut de Biologie, Université de Neuchâtel, Neuchâtel, 2000, Switzerland

[*]Correspondence: despoina.mavridou@austin.utexas.edu

This PDF file includes:

Figures S1 to S4
Tables S1 to S5
Legends for Files S1 to S4
Supplementary references

SUPPLEMENTARY FIGURES

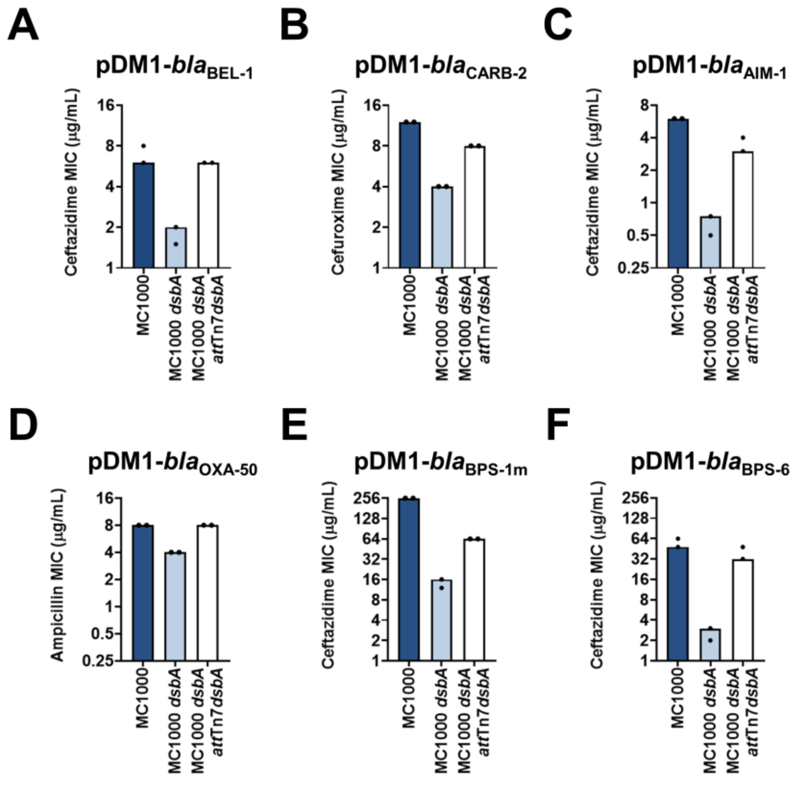

Figure S1. Complementation of *dsbA* restores the β-lactam MIC values for *E. coli* MC1000 *dsbA* expressing β-lactamases. Re-insertion of *dsbA* at the *att*Tn*7* site of the chromosome restores representative β-lactam MIC values for *E. coli* MC1000 *dsbA* harboring **(A)** pDM1-*bla*$_{\text{BEL-1}}$ (ceftazidime MIC), **(B)** pDM1-*bla*$_{\text{CARB-2}}$ (cefuroxime MIC), **(C)** pDM1-*bla*$_{\text{AIM-1}}$ (ceftazidime MIC), **(D)** pDM1-*bla*$_{\text{OXA-50}}$ (ampicillin MIC), **(E)** pDM1-bla$_{\text{BPS-1m}}$ (ceftazidime MIC), and **(F)** pDM1-*bla*$_{\text{BPS-6}}$ (ceftazidime MIC). Graphs show MIC values (µg/mL) and are representative of two biological experiments, each conducted as a single technical repeat.

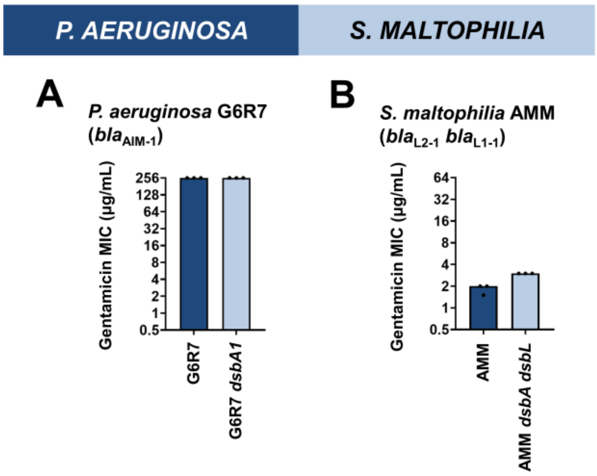

Figure S2. Gentamicin MIC values for representative clinical strains and their *dsbA* mutants. (A) *P. aeruginosa* G6R7 and its mutant lacking *dsbA1* have identical gentamicin MIC values. **(B)** *S. maltophilia* AMM and its mutant lacking *dsbA* and *dsbL* have near-identical gentamicin MIC values. For both organisms, deletion of *dsbA* does not affect the gentamicin MIC values, confirming that absence of DsbA does not compromise their general ability to resist antibiotic stress. Graphs show MIC values (µg/mL) and are representative of three biological experiments, each conducted as a single technical repeat.

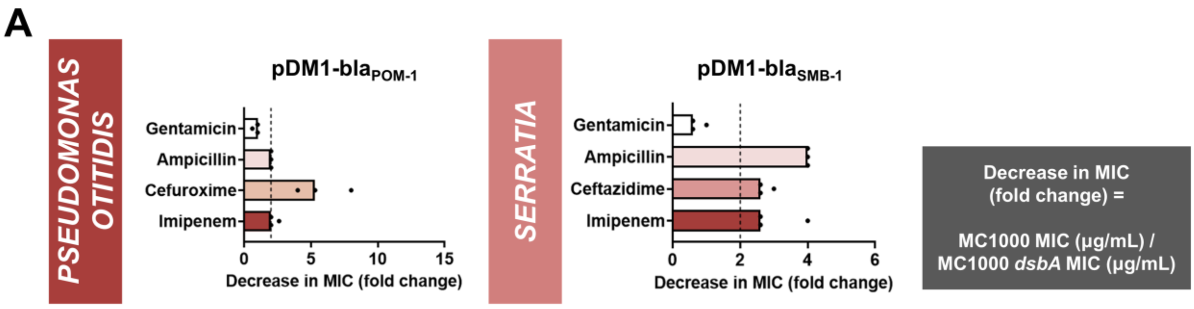

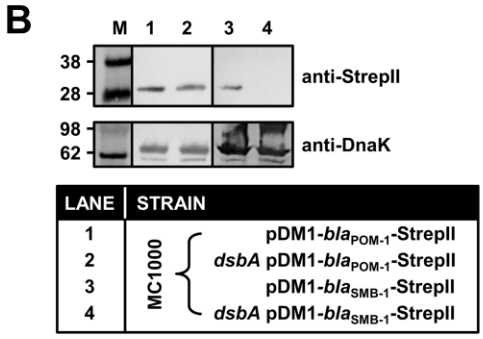

C

Strain (MC1000)	Enzyme stability	Nitrocefin hydrolysis†
pDM1	vector control	1.82 ± 3.20
dsbA pDM1	vector control	0.96 ± 2.06[ns]
pDM1-*bla*$_{L2-1}$	disulfide independent	82.66 ± 1.26
dsbA pDM1-*bla*$_{L2-1}$	disulfide independent	82.99 ± 0.77[ns]
pDM1-*bla*$_{POM-1}$	not degrading	27.87 ± 1.70
dsbA pDM1-*bla*$_{POM-1}$	not degrading	22.23 ± 1.67*
pDM1-*bla*$_{SMB-1}$	degrading	144.96 ± 14.69
dsbA pDM1-*bla*$_{SMB-1}$	degrading	12.11 ± 4.33*

†nM.mg^{-1} pellet.15 min^{-1}

Figure S3. The activity of additional species-specific β-lactamases depends on disulfide bond formation. (A) β-lactam MIC values for *E. coli* MC1000 expressing disulfide-bond-containing β-lactamases from *P. otitidis* (left, POM-1; Table S1) and *Serratia spp*. (right, SMB-1; Table S1) are reduced in the absence of DsbA (MIC fold changes: >2, fold change of 2 is indicated by the black dotted lines). No changes in MIC values are observed for the aminoglycoside antibiotic gentamicin (white bars) confirming that absence of DsbA does not compromise the general ability of this strain to resist antibiotic stress. Graphs show MIC fold changes for β-lactamase-expressing *E. coli* MC1000 and its *dsbA* mutant. MIC assays were performed in three biological experiments each conducted as a single technical repeat; the MIC values used to generate this figure are presented in File S2A (rows 22-25). **(B)** Protein levels of disulfide-bond-containing β-lactamases are either unaffected (POM-1) or drastically reduced (SMB-1) when these enzymes are expressed in *E. coli* MC1000 *dsbA*. Protein levels of StrepII-tagged β-lactamases were assessed using a Strep-Tactin-AP conjugate. A representative blot from three biological experiments, each conducted as a single technical repeat, is shown; molecular weight markers (M) are on the left, DnaK was used as a loading control and solid black lines indicate where the membrane was cut. **(C)** The hydrolytic activities of both tested β-lactamases are significantly reduced in the absence of DsbA. The hydrolytic activities of strains harboring the empty vector or expressing the control enzyme L2-1 show no dependence on DsbA; the data for the control strains are also shown in Figure 2B. n=3 (each conducted in technical duplicate), table shows means ± SD, significance is indicated by * = p < 0.05, ns = non-significant.

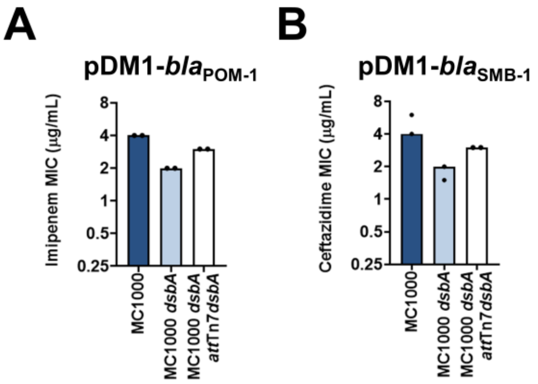

Figure S4. Complementation of *dsbA* restores the β-lactam MIC values for *E. coli* MC1000 *dsbA* expressing β-lactamases. Re-insertion of *dsbA* at the *att*Tn*7* site of the chromosome restores representative β-lactam MIC values for *E. coli* MC1000 *dsbA* harboring **(A)** pDM1-*bla*$_{POM-1}$ (imipenem MIC), and **(B)** pDM1-*bla*$_{SMB-1}$ (ceftazidime MIC). Graphs show MIC values (µg/mL) and are representative of two biological experiments, each conducted as a single technical repeat.

SUPPLEMENTARY TABLES

Table S1. Overview of the β-lactamase enzymes investigated in this study. All tested enzymes belong to distinct phylogenetic clusters (see File S1), with the exception of BPS-1m and BPS-6. The "Cysteine positions" column states the positions of cysteine residues after amino acid 30 and hence, does not include amino acids that are part of the periplasmic signal sequence which is cleaved after protein translocation. All β-lactamase enzymes except L2-1 and LUT-1 (shaded in grey), which are used as negative controls throughout this study, have one or more disulfide bonds. Both L2-1 and LUT-1 contain two or more cysteine residues, but lack disulfide bonds as they are transported to the periplasm in a folded state by the Twin-arginine translocation (Tat) system; for L2-1 Tat-dependent translocation has been experimentally confirmed [1], whereas for LUT-1 this is strongly corroborated by signal peptide prediction software (SignalP 5.0 [2] likelihood scores: Sec/SPI = 0.0572, Tat/SPI = 0.9312, Sec/SPII (lipoprotein) = 0.0087, other = 0.0029). The "Mob." (mobilizable) column refers to the possibility for the β-lactamase gene to be mobilized from the chromosome; "yes" indicates that the gene of interest is located on a mobile element, while "no" refers to immobile chromosomally-encoded enzymes. The "Spectrum" column refers to the hydrolytic spectrum of each tested enzyme; tested enzymes are narrow spectrum β-lactamases (NS), extended spectrum β-lactamases (ESBL) or carbapenemases. The "Inh." (inhibition) column refers to classical inhibitor susceptibility i.e., susceptibility to inhibition by clavulanic acid, tazobactam or sulbactam. Finally, the "Organism" column refers to the bacterial species that most commonly express the tested β-lactamase enzymes.

ENZYME	CYSTEINE POSITIONS	AMBLER CLASS	MOB.	SPECTRUM	INH.	ORGANISM
**L2-1**	C82 C136 C233	A	no	ESBL	yes	*Stenotrophomonas maltophilia*
**LUT-1**	C54 C129	A	no [3]	NS	yes	*Pseudomonas luteola*
**BEL-1**	C61 C231	A	yes [4]	ESBL	yes	*Pseudomonas aeruginosa*
**CARB-2**	C72 C118	A	yes [5]	NS	yes	*Pseudomonas spp.*
**BPS-1m**	C75 C83 C129	A	no [6]	ESBL	yes	*Burkholderia pseudomallei*
**BPS-6**	C75 C83 C129	A	no [7]	ESBL	yes	*Burkholderia pseudomallei*
**GES-19, 20, or 26**	C63 C233	A	yes [8]	ESBLs	yes	*Enterobacteriaceae, Pseudomonas aeruginosa*
**AIM-1**	C31 C56 C194 C199 C234 C274	B3	yes [9]	carbapenemase	no [10]	*Pseudomonas aeruginosa*
**L1-1**	C239 C265	B3	no [10]	carbapenemase	no [10]	*Stenotrophomonas maltophilia*
**POM-1**	C237 C265	B3	no [11]	carbapenemase	no [10]	*Pseudomonas otitidis*
**SMB-1**	C180 C 185 C226 C260	B3	yes [12]	carbapenemase	no [10]	*Serratia spp.*
**OXA-50**	C208 C211	D	no [13]	NS	no [13]	*Pseudomonas spp.*

Table S2. Bacterial strains used in this study. All listed isolates are clinical strains. “FNRCAR” refers to the French National Reference Centre for Antibiotic Resistance in Le Kremlin-Bicêtre, France, and “CDC AR Isolate bank” refers to the Centers for Disease Control and Prevention Antibiotic Resistance Isolate Bank in Atlanta, GA, USA.

NAME	DESCRIPTION	SOURCE
Escherichia coli		
DH5α	F^{-} *end*A1 *gln*V44 *thi*-1 *rec*A1 *rel*A1 *gyr*A96 *deo*R *nup*G *pur*B20 φ80d*lacZ*ΔM15 Δ(*lac*ZYA-*arg*F)U169 *hsd*R17($r_K^- m_K^+$) λ^{-}	[14]
DH5αλpir	λ*pir*	[15]
CC118λpir	*ara*D Δ(*ara, leu*) Δ*lac*Z74 *pho*A20 *gal*K *thi*-1 *rsp*E *rpo*B *arg*E *rec*A1 λ*pir*	[16]
HB101	*sup*E44 *hsd*S20 *rec*A13 *ara*-14 *pro*A2 *lac*Y1 *gal*K2 *rps*L20 *xyl*-5 *mtl*-1	[17]
MC1000	*ara*D139 Δ(*ara, leu*)7697 Δ*lac*X74 *gal*U *gal*K *str*A	[18]
MC1000 *dsbA*	*dsbA::aphA*, Kan^{R}	[19]
MC1000 *dsbA att*Tn*7*::P*tac-dsbA*	*dsbA::aphA att*Tn*7*::*dsbA*, Kan^{R}	[20]
**Clinical isolates**		
Pseudomonas aeruginosa PA14	wild-type prototroph	[21]
Pseudomonas aeruginosa PA14 *att*Tn*7::accC*	*att*Tn*7::accC*, Gent^{R}	This study
Pseudomonas aeruginosa G4R7	*bla*$_{AIM-1}$	FNRCAR
Pseudomonas aeruginosa G4R7 *dsbA1*	*dsbA1 bla*$_{AIM-1}$	This study
Pseudomonas aeruginosa G6R7	*bla*$_{AIM-1}$	FNRCAR
Pseudomonas aeruginosa G6R7 *dsbA1*	*dsbA1 bla*$_{AIM-1}$	This study
Pseudomonas aeruginosa CDC #769	*bla*$_{GES-19}$ *bla*$_{GES-26}$	CDC AR Isolate Bank
Pseudomonas aeruginosa CDC #769 *dsbA1*	*dsbA1 bla*$_{GES-19}$ *bla*$_{GES-26}$	This study
Pseudomonas aeruginosa CDC #773	*bla*$_{GES-19}$ *bla*$_{GES-20}$	CDC AR Isolate Bank
Pseudomonas aeruginosa CDC #773 *dsbA1*	*dsbA1 bla*$_{GES-19}$ *bla*$_{GES-20}$	This study
Stenotrophomonas maltophilia AMM	*bla*$_{L2-1}$ *bla*$_{L1-1}$	[22]
Stenotrophomonas maltophilia AMM *dsbA dsbL*	*dsbA dsbL bla*$_{L2-1}$ *bla*$_{L1-1}$	This study
Stenotrophomonas maltophilia GUE	*bla*$_{L2-1}$ *bla*$_{L1-1}$	[22]
Stenotrophomonas maltophilia GUE *dsbA dsbL*	*dsbA dsbL bla*$_{L2-1}$ *bla*$_{L1-1}$	This study

Table S3. Plasmids used in this study.

NAME	DESCRIPTION	SOURCE
pDM1	pDM1 vector (GenBank MN128719), p15A *ori*, P*tac* promoter, MCS, Tet^R	Mavridou lab stock
pDM1-*bla*$_{L2\text{-}1}$	*bla*$_{L2\text{-}1}$ cloned into pDM1, Tet^R	[20]
pDM1-*bla*$_{LUT\text{-}1}$	*bla*$_{LUT\text{-}1}$ cloned into pDM1, Tet^R	This study
pDM1-*bla*$_{BEL\text{-}1}$	*bla*$_{BEL\text{-}1}$ cloned into pDM1, Tet^R	This study
pDM1-*bla*$_{CARB\text{-}2}$	*bla*$_{CARB\text{-}2}$ cloned into pDM1, Tet^R	This study
pDM1-*bla*$_{BPS\text{-}1m}$	*bla*$_{BPS\text{-}1m}$ cloned into pDM1, Tet^R	This study
pDM1-*bla*$_{BPS\text{-}6}$	*bla*$_{BPS\text{-}6}$ cloned into pDM1, Tet^R	This study
pDM1-*bla*$_{AIM\text{-}1}$	*bla*$_{AIM\text{-}1}$ cloned into pDM1, Tet^R	This study
pDM1-*bla*$_{POM\text{-}1}$	*bla*$_{POM\text{-}1}$ cloned into pDM1, Tet^R	This study
pDM1-*bla*$_{SMB\text{-}1}$	*bla*$_{SMB\text{-}1}$ cloned into pDM1, Tet^R	This study
pDM1-*bla*$_{OXA\text{-}50}$	*bla*$_{OXA\text{-}50}$ cloned into pDM1, Tet^R	This study
pDM1-*bla*$_{BEL\text{-}1}$-StrepII	*bla*$_{BEL\text{-}1}$ encoding BEL-1 with a C-terminal StrepII tag cloned into pDM1, Tet^R	This study
pDM1-*bla*$_{CARB\text{-}2}$-StrepII	*bla*$_{CARB\text{-}2}$ encoding CARB-2 with a C-terminal StrepII tag cloned into pDM1, Tet^R	This study
pDM1-*bla*$_{BPS\text{-}1m}$-StrepII	*bla*$_{BPS\text{-}1m}$ encoding BPS-1m with a C-terminal StrepII tag cloned into pDM1, Tet^R	This study
pDM1-*bla*$_{AIM\text{-}1}$-StrepII	*bla*$_{AIM\text{-}1}$ encoding AIM-1 with a C-terminal StrepII tag cloned into pDM1, Tet^R	This study
pDM1-*bla*$_{L1\text{-}1}$-StrepII	*bla*$_{L1\text{-}1}$ encoding L1-1 with a C-terminal StrepII tag cloned into pDM1, Tet^R	[20]
pDM1-*bla*$_{POM\text{-}1}$-StrepII	*bla*$_{POM\text{-}1}$ encoding POM-1 with a C-terminal StrepII tag cloned into pDM1, Tet^R	This study
pDM1-*bla*$_{SMB\text{-}1}$-StrepII	*bla*$_{SMB\text{-}1}$ encoding SMB-1 with a C-terminal StrepII tag cloned into pDM1, Tet^R	This study
pDM1-*bla*$_{OXA\text{-}50}$-StrepII	*bla*$_{OXA\text{-}50}$ encoding OXA-50 with a C-terminal StrepII tag cloned into pDM1, Tet^R	This study
pKNG101	Gene replacement suicide vector, *ori*R6K, *oriT*RK2, *sacB*, Str^R	[23]
pKNG102	Gene replacement suicide vector, *ori*R6K, *oriT*RK2, *sacB*, Tet^R	Bernal lab stock
pKNG101-*dsbA1*	PCR fragment containing the regions upstream and downstream *P. aeruginosa* PA43417 [24] *dsbA1* cloned in pKNG101; when inserted into the chromosome, the strain is a merodiploid for *dsbA1* mutant, Str^R	[20]
pKNG102-*dsbA1*-769	PCR fragment containing the regions upstream and downstream *P. aeruginosa* CDC #769 (Table S2) *dsbA1* cloned in pKNG102; when inserted into the chromosome, the strain is a merodiploid for *dsbA1* mutant, Tet^R	This study
pKNG102-*dsbA1*-773	PCR fragment containing the regions upstream and downstream *P. aeruginosa* CDC #773 (Table S2) *dsbA1* cloned in	This study

	pKNG102; when inserted into the chromosome, the strain is a merodiploid for *dsbA1* mutant, Tet^R	
pKNG101-*dsbA dsbL*-AMM	PCR fragment containing the regions upstream and downstream *S. maltophilia* AMM *dsbA* and *dsbL* genes cloned in pKNG101; when inserted into the chromosome, the strain is a merodiploid for *dsbA dsbL* mutant, Str^R	This study
pKNG101-*dsbA dsbL*-GUE	PCR fragment containing the regions upstream and downstream *S. maltophilia* GUE *dsbA* and *dsbL* genes cloned in pKNG101; when inserted into the chromosome, the strain is a merodiploid for *dsbA dsbL* mutant, Str^R	This study
pRK600	Helper plasmid, ColE1 *ori*, *mob*RK2, *tra*RK2, Cam^R	[25]
pTn*7*-M	Mini-Tn*7* delivery transposon vector containing the Tn*7* flanking regions and a $Gent^R$ marker, R6K *ori*, Kan^R, $Gent^R$	[26]
pTNS2	Helper plasmid, R6K *ori*; encodes the TnsABC+D specific transposition pathway, Amp^R	[27]
pMK-RQ *carb-2*	GeneArt® cloning vector containing *carb-2*, ColE1 *ori*, (template for *carb-2*), Kan^R	This study
pMK-RQ *bps-1m*	GeneArt® cloning vector containing *bps-1m*, ColE1 *ori*, (template for *bps-1m*), Kan^R	This study
pMK-RQ *bps-6*	GeneArt® cloning vector containing *bps-6*, ColE1 *ori*, (template for *bps-6*), Kan^R	This study
pMK-RQ *smb-1*	GeneArt® cloning vector containing *smb-1*, ColE1 *ori*, (template for *smb-1*), Kan^R	This study

Table S4. Oligonucleotide primers used in this study. The "Brief description" column provides basic information on the primer design (restriction enzyme used for cloning, encoded protein or gene replaced by antibiotic resistance cassette, forward or reverse orientation of the primer (F or R); SQ stands for sequencing primers).

NUMBER	BRIEF DESCRIPTION	SEQUENCE (5´-3´)
P1	SacI.LUT-1.F	ctggagctcaatgtcatcctgaaccgtcga
P2	PstI.LUT-1.R	cagctgcagtcagcctgtcacccattcag
P3	SacI.BEL-1.F	ctggagctcaaactgctctacccgttattgc
P4	PstI.BEL-1.R	cagctgcagtcagtgaacatattgacgtgc
P5	SacI.CARB-2.F	ctggagctcaagtttttattggcattttcgc
P6	KpnI.CARB-2.R	cagggtacctcagcgcgactgtgatgta
P7	SacI.BPS-1m.F	ctggagctcaatcattctccgttgcgccgctc
P8	XmaI.BPS-1m.R	caacccgggtcaggcgaacgcccgcgcg
P9	SacI.AIM-1.F	ctggagctcaaacgtcgcttcaccctgg
P10	KpnI.AIM-1.R	ctgggtacctcaaggccgcgcgccgctg
P11	SacI.POM-1.F	ctggagctccgtaccctgaccctcg
P12	KpnI.POM-1.R	cagggtaccttatgcgtcatcagagacctc
P13	NdeI.SMB-1.F	cagctccatatgaaaatcatcgcttccctgatcc
P14	XmaI.SMB-1.R	ctgcccgggtcagcgtttctcgctggcca
P15	SacI.OXA-50.F	ctggagctccgccctctcttcagtg
P16	KpnI.OXA-50.R	cagggtacctcagggcagtatcccgagag
P17	PstI.StrepII.BEL-1.R	cagctgcagttatttttcaaattgcggatggctccaagcgctc ccgtgaacatattgacgtgctaac
P18	KpnI.StrepII.CARB-2.R	cagggtaccttatttttcaaattgcggatggctccaagcgctc ccgcgcgactgtgatgtataa
P19	XmaI.StrepII.BPS-1m.R	ctgcccgggctatttttcaaattgcggatggctccaagcgct cccggcgaacgcccgcgcggcg
P20	KpnI.StrepII.AIM-1.R	cagggtaccttatttttcaaattgcggatggctccaagcgctc ccaggccgcgcgccgctggag
P21	KpnI.StrepII.POM-1.R	cagggtaccttatttttcaaattgcggatggctccaagcgctc ccgccgcgctgcttc
P22	XmaI.StrepII.SMB-1.R	ctgcccgggctatttttcaaattgcggatggctccaagcgct cccgcgtttctcgctggccag
P23	KpnI.StrepII.OXA-50.R	cagggtaccttatttttcaaattgcggatggctccaagcgctc ccgggcagtatcccgagagcc
P24	SQ.dsbA1.Paeruginosa.F	tacctgctcaagcagatgcatg
P25	SQ.dsbA1.Paeruginosa.R	ggtgttcatgtcgcccatca
P26	SQ.dsbAdsbL.Smaltophilia.F	atggtgccgttcgtgcaga
P27	SQ.dsbAdsbL.Smaltophilia.R	acagcacctgcatttccgg
P28	XbaI.dsbA1.F	ggttcctctagagcctacttcgccagccagaa
P29	pKNG101-dsbA1.body.R	ctacttcttgttacgcatcgttcactc
P30	pKNG101-dsbA1.body.F	atgcgtaacaagaagtaggcaaggtga
P31	BamHI.dsbA1.R	aattaaggatcctcatcactaccaccagcgcg
P32	XbaI.dsbAdsbL.F	ggttcctctagatcttctggtacagcacctgcatttccg
P33	pKNG102-dsbAdsbL.body.R	tgcgtgtcgatgaggttggctcactga
P34	pKNG102-dsbAdsbL.body.F	tctcttggatcagtgagccaacctcat
P35	BamHI.dsbAdsbL.R	aattaaggatcctcgctggaggtggatttcagcaagacc

Table S5. Sources of genomic DNA used for amplification of β-lactamase genes used in this study. CRBIP stands for Centre de Ressources Biologiques de l'Institut Pasteur, France and FNRCAR refers to the French National Reference Centre for Antibiotic Resistance in Le Kremlin-Bicêtre, France.

STRAIN	GENE	SOURCE
Pseudomonas aeruginosa 51170	$bla_{\mathrm{BEL-1}}$	[4]
Pseudomonas luteola CIP 102067	$bla_{\mathrm{LUT-1}}$	CRBIP
Pseudomonas aeruginosa G4R7	$bla_{\mathrm{AIM-1}}$	FNRCAR
Pseudomonas otitidis CIP 109236T	$bla_{\mathrm{POM-1}}$	CRBIP
Pseudomonas aeruginosa PAO1 LA	$bla_{\mathrm{OXA-50}}$	[28]

LEGENDS FOR SUPPLEMENTARY DATA FILES

File S1. Analysis of the cysteine content and phylogeny of all identified β-lactamases. 7,233 unique β-lactamase protein sequences were clustered with a 90% identity threshold and the centroid of each cluster was used as a phylogenetic cluster identified for each sequence ("Phylogenetic cluster" column). All sequences were searched for the presence of cysteine residues ("Total number of cysteines" and "Positions of all cysteines" columns). Proteins with two or more cysteines after the first 30 amino acids of their primary sequence (cells shaded in grey in the "Number of cysteines after position 30" column) are potential substrates of the DSB system for organisms where oxidative protein folding is carried out by DsbA and provided that translocation of the β-lactamase outside the cytoplasm is performed by the Sec system. The first 30 amino acids of each sequence were excluded to avoid considering cysteines that are part of the signal sequence mediating the translocation of these enzymes outside the cytoplasm. Cells shaded in grey in the "Reported in pathogens" column mark β-lactamases that are found in pathogens or organisms capable of causing opportunistic infections. The Ambler class of each enzyme is indicated in the "Ambler class column" and each class (A, B1, B2, B3, C and D) is highlighted in a different color.

File S2. Data used to generate Figure 1, Figure S4, and Figure 5B. (A) MIC values (μg/mL) used to generate Figure 1 are in rows 2-7 [strains serving as negative controls; *E. coli* MC1000 strains harboring pDM1 (vector alone), pDM1-bla_{L2-1} or pDM1-bla_{LUT-1} (cysteine-containing β-lactamases which lack disulfide bonds)] and rows 9-20. MIC values (μg/mL) used to generate Figure S4 are in rows 22-25. The aminoglycoside antibiotic gentamicin serves as a negative control for all strains. Cells marked with a dash (-) represent strain-antibiotic combinations that were not tested. **(B)** *P. aeruginosa* PA14 CFU counts used to generate Figure 5B. For both tabs, three biological experiments are shown; for (B) each biological replicate was conducted in technical triplicate and mean CFU values are shown.

File S3. Analysis of *Stenotrophomonas spp.* for the presence of MCR proteins. Hidden Markov Models built from validated sequences of MCR-like and EptA-like proteins were used for the identification of MCR-like analogues in a total of 106 complete genomes of the *Stenotrophomonas* genus downloaded from the NCBI repository. **(A)** All genomes that were investigated ("*Stenotrophomonas maltophilia* genome" column"), except for one, encoded one or two MCR-like proteins ("Number of MCR analogues column"). **(B)** The 146 MCR-like sequences ("Protein ID column") that were identified (only hits with evalues < 1e-10 were considered; "Evalue" column) belong to the same phylogenetic group as validated MCR-5 or MCR-8 proteins ("Phylogenetic group" column).

File S4. Quality control information on 4,5-dibromo-2-(2-chlorobenzyl)pyridazin-3(2H)-one. ^{1}H-NMR and LCMS spectra of 4,5-dibromo-2-(2-chlorobenzyl)pyridazin-3(2H)-one (compound 36) demonstrating the correctness and purity of the synthesized compound by Bioduro-Sundia.

SUPPLEMENTARY REFERENCES

1. Pradel N, Delmas J, Wu LF, Santini CL, Bonnet R (2009) Sec- and Tat-dependent translocation of β-lactamases across the *Escherichia coli* inner membrane. Antimicrob. Agents Chemother. 53: 242-248.

2. Almagro Armenteros JJ, Tsirigos KD, Sonderby CK, Petersen TN, Winther O, et al. (2019) SignalP 5.0 improves signal peptide predictions using deep neural networks. Nat. Biotechnol. 37: 420-423.

3. Doublet B, Robin F, Casin I, Fabre L, Le Fleche A, et al. (2010) Molecular and biochemical characterization of the natural chromosome-encoded class A β-lactamase from *Pseudomonas luteola*. Antimicrob. Agents Chemother. 54: 45-51.

4. Poirel L, Brinas L, Verlinde A, Ide L, Nordmann P (2005) BEL-1, a novel clavulanic acid-inhibited extended-spectrum β-lactamase, and the class 1 integron In120 in *Pseudomonas aeruginosa*. Antimicrob. Agents Chemother. 49: 3743-3748.

5. Bert F, Branger C, Lambert-Zechovsky N (2002) Identification of PSE and OXA β-lactamase genes in *Pseudomonas aeruginosa* using PCR-restriction fragment length polymorphism. J. Antimicrob. Chemother. 50: 11-18.

6. Ho PL, Cheung TKM, Yam WC, Yuen KY (2002) Characterization of a laboratory-generated variant of BPS β-lactamase from *Burkholderia pseudomallei* that hydrolyses ceftazidime. J. Antimicrob. Chemother. 50: 723-726.

7. Tribuddharat C, Moore RA, Baker P, Woods DE (2003) *Burkholderia pseudomallei* class a β-lactamase mutations that confer selective resistance against ceftazidime or clavulanic acid inhibition. Antimicrob. Agents Chemother. 47: 2082-2087.

8. Yoon EJ, Jeong SH (2021) Mobile carbapenemase genes in *Pseudomonas aeruginosa*. Front. Microbiol. 12: 614058.

9. Yong D, Toleman MA, Bell J, Ritchie B, Pratt R, et al. (2012) Genetic and biochemical characterization of an acquired subgroup B3 metallo-β-lactamase gene, $\textit{bla}_{\text{AIM-1}}$, and its unique genetic context in *Pseudomonas aeruginosa* from Australia. Antimicrob. Agents Chemother. 56: 6154-6159.

10. Tooke CL, Hinchliffe P, Bragginton EC, Colenso CK, Hirvonen VHA, et al. (2019) Β-lactamases and β-lactamase inhibitors in the 21st century. J. Mol. Biol. 431: 3472-3500.

11. Thaller MC, Borgianni L, Di Lallo G, Chong Y, Lee K, et al. (2011) Metallo-β-lactamase production by *Pseudomonas otitidis*: a species-related trait. Antimicrob. Agents Chemother. 55: 118-123.

12. Wachino J, Yoshida H, Yamane K, Suzuki S, Matsui M, et al. (2011) SMB-1, a novel subclass B3 metallo-β-lactamase, associated with ISCR1 and a class 1 integron, from a carbapenem-resistant *Serratia marcescens* clinical isolate. Antimicrob. Agents Chemother. 55: 5143-5149.

13. Girlich D, Naas T, Nordmann P (2004) Biochemical characterization of the naturally occurring oxacillinase OXA-50 of *Pseudomonas aeruginosa*. Antimicrob. Agents Chemother. 48: 2043-2048.

14. Hanahan D (1985) In: Glover DM, editor. DNA Cloning: A Practical Approach: IRL Press, McLean, Virginia. pp. 109.

15. Martinez-Garcia E, de Lorenzo V (2011) Engineering multiple genomic deletions in Gram-negative bacteria: analysis of the multi-resistant antibiotic profile of *Pseudomonas putida* KT2440. Environ. Microbiol. 13: 2702-2716.

16. Herrero M, de Lorenzo V, Timmis KN (1990) Transposon vectors containing non-antibiotic resistance selection markers for cloning and stable chromosomal insertion of foreign genes in Gram-negative bacteria. J. Bacteriol. 172: 6557-6567.

17. Boyer HW, Roulland-Dussoix D (1969) A complementation analysis of the restriction and modification of DNA in *Escherichia coli*. J. Mol. Biol. 41: 459-472.

18. Casadaban MJ, Cohen SN (1980) Analysis of gene control signals by DNA fusion and cloning in *Escherichia coli*. J. Mol. Biol. 138: 179-207.

19. Kadokura H, Tian H, Zander T, Bardwell JC, Beckwith J (2004) Snapshots of DsbA in action: detection of proteins in the process of oxidative folding. Science 303: 534-537.

20. Furniss RCD, Kaderabkova N, Barker D, Bernal P, Maslova E, et al. (2022) Breaking antimicrobial resistance by disrupting extracytoplasmic protein folding. eLife 11.

21. Rahme LG, Stevens EJ, Wolfort SF, Shao J, Tompkins RG, et al. (1995) Common virulence factors for bacterial pathogenicity in plants and animals. Science 268: 1899-1902.

22. Emeraud C, Escaut L, Boucly A, Fortineau N, Bonnin RA, et al. (2019) Aztreonam plus clavulanate, tazobactam, or avibactam for treatment of infections caused by metallo-β-lactamase -producing Gram-negative bacteria. Antimicrob. Agents Chemother. 63.

23. Kaniga K, Delor I, Cornelis GR (1991) A wide-host-range suicide vector for improving reverse genetics in Gram-negative bacteria: inactivation of the *blaA* gene of *Yersinia enterocolitica*. Gene 109: 137-141.

24. El Garch F, Bogaerts P, Bebrone C, Galleni M, Glupczynski Y (2011) OXA-198, an acquired carbapenem-hydrolyzing class D β-lactamase from *Pseudomonas aeruginosa*. Antimicrob. Agents Chemother. 55: 4828-4833.

25. Kessler B, Delorenzo V, Timmis KN (1992) A general system to integrate *lacZ* fusions into the chromosomes of Gram-negative eubacteria: regulation of the *Pm* Promoter of the *TOL* plasmid studied with all controlling elements in monocopy. Mol. Gen.Genet. 233: 293-301.

26. Zobel S, Benedetti I, Eisenbach L, de Lorenzo V, Wierckx N, et al. (2015) Tn*7*-based device for calibrated heterologous gene expression in *Pseudomonas putida*. ACS Synth. Biol. 4: 1341-1351.

27. Choi KH, Gaynor JB, White KG, Lopez C, Bosio CM, et al. (2005) A Tn*7*-based broad-range bacterial cloning and expression system. Nat. Methods 2: 443-448.

28. Holloway BW (1969) Genetics of *Pseudomonas*. Bacteriol. Rev. 33: 419-443.
